# Botulinum Toxin in the Treatment of Headache

**DOI:** 10.3390/toxins12120803

**Published:** 2020-12-17

**Authors:** Werner J. Becker

**Affiliations:** Department of Clinical Neurosciences, Foothills Medical Centre, University of Calgary, 1403 29th St. NW, Calgary, AB T2N 2T9, Canada; wbecker@ucalgary.ca

**Keywords:** chronic migraine, migraine, botulinum toxin, onabotulinumtoxinA, erenumab, CGRP

## Abstract

Botulinum toxin type A has been used in the treatment of chronic migraine for over a decade and has become established as a well-tolerated option for the preventive therapy of chronic migraine. Ongoing research is gradually shedding light on its mechanism of action in migraine prevention. Given that its mechanism of action is quite different from that of the new monoclonal antibodies directed against calcitonin gene-related peptide (CGRP) or its receptor, it is unlikely to be displaced to any major extent by them. Both will likely remain as important tools for patients with chronic migraine and the clinicians assisting them. New types of botulinum toxin selective for sensory pain neurons may well be discovered or produced by recombinant DNA techniques in the coming decade, and this may greatly enhance its therapeutic usefulness. This review summarizes the evolution of botulinum toxin use in headache management over the past several decades and its role in the preventive treatment of chronic migraine and other headache disorders.

## 1. Introduction

After the approval of onabotulinumtoxinA (OBTA) for prophylaxis of chronic migraine in 2010 in the USA, and in 2011 in Canada, it became a mainstream therapy for chronic migraine. It was clearly as effective if not more effective than any other migraine preventive medication available at the time and proved to be well tolerated.

The use of botulinum toxin (BT) in the treatment of headache in North America is dominated by OBTA, the Allergan botulinum toxin type A (BTA) compound, although it was not given that name until 2009. There has been minimal research with other BTs in migraine. An open-label study with 63 patients utilizing incobotulinumtoxinA (Xeomin) showed benefit [1], and a retrospective study involving 128 patients who received rimabotulinumtoxinB (Myobloc) was also positive for benefit [2]. From a global perspective, several other BTs, mainly BTAs, have been developed in South Korea, China, and Russia [3]. Although all BTAs have the same mode of action, there are differences between them including how potency units are measured, so they are not interchangeable [4].

OBTA remains a mainstay of chronic migraine treatment, but with the advent of the calcitonin gene-related peptide (CGRP)-related monoclonal antibodies in the USA and Canada in 2018, the practice milieu shifted considerably. The place of OBTA in migraine prevention in the future remains to be determined.

## 2. Chronic Migraine

OBTA is indicated for chronic migraine. The diagnostic criteria for chronic migraine include headache on at least 15 days a month, and eight of these headache days must be migraine headaches or relieved by a triptan or ergot derivative. [5]. The importance of effective migraine therapy cannot be overemphasized. At level four in the 2016 Global Burden of Disease Study, migraine was estimated to be responsible for 5.6% of all years of life lived with disability and ranked second among medical disorders, behind only low back pain [6]. Individuals with chronic migraine occupy the more severe end of the migraine-related disability spectrum.

## 3. History of Botulinum Toxin

The scientific history of BT began in 1820 when Justinus Kerner published a description of botulism. Professor van Ermengem at the University of Ghent isolated the causative organism in 1897 and named it Bacillus botulinum, a name later changed to Clostridium botulinum [7].

The clinical use of BT began when Alan Scott used it in strabismus in 1977. He obtained FDA approval in 1989 for BTA (Oculinum) to treat strabismus, blepharospasm, and hemifacial spasm [7]. Over 150 years had gone by from the time of Kerner’s observations to the clinical use of BT.

## 4. Early Use of Botulinum Toxin in Headache

During the 1990s, there was increasing interest in treating tension-type headache with BT because of its muscle-paralytic actions. In the first publication on this topic in 1994, Sjaastad’s group in Norway reported a negative trial with BTA injected into the temporal muscles [8].

A case report in 1998 noted that frontal and periorbital headaches were relieved in a patient treated with BTA for blepharospasm [9], and in the same year, a publication on the use of BTA in tension-type headache reported it reduced the severity and frequency of migraine-type headaches [10].

In 1999, a case series from Germany reported that injections of BTA into the muscles of the head and neck alleviated tension-type headache [11], and a case series from Canada concluded that BTA used for wrinkles was helpful in reducing tension-type headache [12].

There was now so much interest in using one of the most potent poisons known to mankind to treat headache that the title of a paper in the Harvard Health Letter read “Feed a cold, starve a fever, and poison a headache” [13].

Interest in using BTA for migraine increased, and in 2000, Binder reported benefit for migraine in an open-label study [14]. Allergan, which had obtained the rights to Oculinum from Scott in 1991 and rebranded it as Botox, launched a long-term clinical trials program for testing BTA in headache.

## 5. Modern Clinical Trials in Migraine

Modern clinical trials of BTA in migraine began with a small randomized double-blind placebo-controlled (RDBPC) trial in 2000. Most patients had episodic migraine, and the doses of BTA in the two active treatment groups were small: 25 and 75 units. It yielded a paradoxical result. The group receiving 25 units showed a statistically significant difference from placebo for headache frequency and severity, while the 75-unit group did not [15]. Nevertheless, this trial, in combination with the previous open-label trials and case reports, initiated a massive clinical research program into the possible benefits of BTA in migraine.

By 2002, some headache specialists were already stating that “Clinical trials, retrospective studies, and case studies indicate that BTA is safe and effective for prophylactic and acute treatment of migraine headache” [16]. Despite this, it would be a long time before BTA had accumulated enough evidence to obtain FDA approval, and approval was granted only for preventive treatment of chronic migraine.

Two large RDBPC trials in patients with “chronic daily headache” followed: one with 355 subjects and one with 702. In both, the primary endpoint was not met, but some secondary endpoints suggested efficacy. In the Mathew study, where subjects received approximately 190 units of BTA over the forehead, scalp, neck, and trapezius muscles, the percentage of patients with a ≥50% reduction in headache days per month was greater in the BTA group versus placebo (32.7 vs. 15.0%, *p* = 0.027) [17]. The larger Silberstein study was even less conclusive, but again, there was some indication of benefit [18]. These trials led to the larger Phase III Research Evaluating Migraine Prophylaxis Therapy (PREEMPT) trials, which randomized 1384 subjects [19,20].

The PREEMPT trials finally established Allergan’s BTA, now known as OBTA, as an efficacious preventive therapy for chronic migraine, but there was still controversy.

In PREEMPT I [19], the primary endpoint, mean change in headache episodes from baseline, was negative. However, several secondary endpoints were positive, including a reduction in headache days and migraine days. Based on these results, the primary endpoint in PREEMPT II [20], which had not yet been analyzed, was changed from reduction in headache episodes to reduction in headache days per month. PREEMPT II met its new primary endpoint.

The magnitude of the placebo response in the PREEMPT studies relative to the difference between placebo and OBTA also caused concern. In PREEMPT II, for example, headache days per month in the placebo groups were reduced from a baseline of 19.7 days to 13 days at month 6, a difference of 6.7 days. The active treatment group that received OBTA showed a reduction of 9 headache days per month, thereby exceeding the placebo response by 2.3 days. This difference appeared small to some but the difference in headache hours per month between OBTA and placebo was 42.4 h, which equates for many to an average work week. In PREEMPT I, the reduction in headache hours per month was similar, with the OBTA group showing a reduction in headache hours of 36.3 h per month over that found in the placebo group. The Headache Impact Test—6 and the Migraine-Specific Quality of Life questionnaire both showed statistically significant greater improvement in the OBTA group compared to the placebo group [21]. Additional analyses showed that chronic migraine patients with medication overuse headache and those without overuse did equally well with OBTA.

There was also concern that blinding might have been incomplete, since OBTA can cause muscle weakness, although this would be evident primarily in the frontal and glabellar regions. The PREEMPT studies did not assess the blinding, but the older Mathew study, which had used similar doses of OBTA, found that after the second BTA treatment, 65% of subjects correctly guessed the treatment received. After the third treatment, only 60% guessed correctly [17]. By chance alone, 50% of patients should have guessed correctly, so blinding appeared to have been largely maintained.

The generalizability of the PREEMPT studies could be criticized in that all subjects needed to have some headache free time to be included. At least four distinct headache episodes per month were required. The COMPEL study, a large long-term open-label study funded by Allergan, further explored the type of chronic migraine patient who responded to OBTA. It found that in the long term, patients with daily headache had a very similar reduction in the number of moderate or severe headache days per month as patients without daily headache at baseline [22].

Ironically, the apparently positive results in the early open-label study by Binder [14] and the small RDBPC trial by Silberstein [15], which provided the impetus for the later large RDBPC trials, were likely due to the placebo effect and/or random error. These trials consisted mainly of patients with episodic migraine, and a much larger later episodic migraine RDBPC trial in 2007 [23], which randomized 369 subjects, showed no significant difference between BTA and placebo. There was suggestion of benefit in a post hoc analysis in patients with a headache frequency of 12 days a month or more.

OBTA was eventually approved in many countries for chronic migraine only.

## 6. Mechanism of Action

The clinical observation that in some people OBTA injected for cosmetic purposes appeared to alleviate headache led to the large clinical trials program that eventually made OBTA available for the treatment of chronic migraine. Much of the research into how it produced improvement in chronic migraine came later. OBTA is administered in the scalp and migraine pain is thought to come from the meninges, but research has gradually uncovered the mechanisms likely involved.

### 6.1. Action of OBTA at the Cellular Level

When OBTA is injected into the extracellular space, the heavy chain of the toxin binds to receptors on the C-fiber nerve terminal, OBTA is endocytosed and enters the nerve terminal enclosed in a vesicle. The light chain then dissociates from the heavy chain and enters the cell cytoplasm where it cleaves SNAP-25 (synaptosomal-associated protein), a protein critical for fusion of neuropeptide-bearing vesicles with the nerve terminal membrane. As a result, CGRP and other neuropeptides cannot be released. The insertion of some receptors that are brought to the cell membrane by vesicles (e.g., TRPV1, P2X3) is also blocked [24].

When C-fiber sensory nerve endings in the meninges are activated by stimuli, they release neuropeptides including CGRP. The ability of OBTA to block CGRP release from peripheral C-fiber nerve endings is likely critical to its therapeutic role in migraine, although inhibition of neuropeptide and neurotransmitter release in the trigeminal ganglion and possibly in the central terminals of the peripheral sensory neurons may also play a role [24,25,26]. Inhibition of insertion of certain receptors (e.g., TRPV1 and TRPA1) into nociceptor cell membranes may also be important [27].

### 6.2. Mechanisms for Extracranial OBTA to Affect Pain from the Dura

The demonstration that some sensory neurons have branches that terminate on the dura and other branches that leave the cranium through sutures and emissary canals to terminate in the scalp provides a potential explanation for how scalp-injected OBTA could influence intracranial nociception [28]. The observation that extracranial administration of OBTA, at least in the rat, inhibits C-fibers responses to stimulation of their intracranial receptive fields with ligands of TRPV1 and TRPA1 channels is in keeping with the above anatomical observations [27]. It could also be speculated that OBTA helps to relieve migraine pain in some patients directly through reduction in nociceptive activity in extracranial nerves or through reduction in circulating CGRP. OBTA reduces blood CGRP levels in patients with chronic migraine who respond to it with a reduction in headache frequency [29].

### 6.3. Blocking the Migraine Attack

The pathophysiology of migraine is complex and still not completely understood [30]. An important feature in the genesis of a migraine attack in some patients may be the activation of C-fiber nociceptors in the dura. In migraine with aura, this activation may occur because of diffusion to the meninges of substances released from the cerebral cortex during spreading depression [31,32]. When activated, these nerve endings release CGRP, which sensitizes the C-fiber nociceptors further and may activate adjacent A-delta pain fibers [33,34]. Afferent activity from these two nerve fiber types flows to the trigeminocervical complex (TCC) in the brainstem, where second order neurons in the trigeminal sensory pathways are activated. It has also been proposed that migraine headache pain may result from dysfunctional CGRP signaling in the trigeminal ganglion [35].

OBTA may interrupt this sequence of events by inhibiting the release of CGRP and other neuropeptides from the nociceptive C-fibers. This would not only prevent the C-fiber nociceptor from becoming sensitized but would also prevent the activation of nearby A-delta fibers by CGRP released from the C-fiber nerve terminals [33]. Thus, the afferent flow into the TCC would be greatly reduced.

## 7. Clinical Use

Since OBTA was approved for use in chronic migraine in the USA in 2010 and in Canada in 2011, it has become widely used in migraine attack prevention. The PREEMPT studies provided a proven injection paradigm for its use in chronic migraine, but it required a minimum of 31 injections of 5 units each into specific points in the glabellar, frontal, temporal, occipital, upper cervical, and trapezius regions. In addition, depending upon the location of the head pain or tender points, an additional eight injections could be administered in specific regions (temporal, occipital, and trapezius) as a “follow the pain” option. The dosage of OBTA could vary from 155 units (fixed site injections only) to 195 units (if all the optional “follow the pain” injections were added) [36].

Injection technique was important, as poor technique could increase the risks of local side effects including eyelid ptosis, brow ptosis, neck pain, neck weakness, and shoulder pain. The PREEMPT protocol was refined over time to reduce the occurrence of these side effects [37]. It is of interest that the US label for Botox specifies 155 U at 31 injection sites, while the labels in many other countries including Canada are more consistent with the original PREEMPT protocol and allow up to 195 U and 39 injection sites [38]. However, a survey has shown that 77% of American headache clinicians modify the PREEMPT protocol as defined in the US label and use more injections sites and more than 155 units. Some injection sites not included in the PREEMPT protocol, for example masseter injections, are also used [39].

The benefits of OBTA in patients with chronic migraine tend to persist. A study of 108 patients who responded to OBTA found that only a small minority lost their response to treatment over the next several years [40].

There is also evidence that patients with chronic migraine who are treated earlier in their clinical course are more likely to ultimately be able to discontinue OBTA and maintain a prolonged remission, suggesting that early treatment of patients may be important for better outcomes [41].

## 8. Current Place in Therapy

Several treatment guidelines have recognized the role that OBTA can play in chronic migraine therapy. In 2016, the American Academy of Neurology recommended that OBTA “should be offered as a treatment option to patients with CM to increase the number of headache-free days (Level A) and should be considered to reduce headache impact on health-related QOL (Level B)” [42]. In 2018, the European Headache Federation stated in its guideline: “OnabotulinumtoxinA is recommended for treatment of patients with CM and considered an effective and well tolerated treatment. Quality of evidence: high. Strength of the recommendation: strong.” Even though it was acknowledged that there is evidence that shorter disease duration may lead to a better response to OBTA, it still recommended, based purely on expert opinion, that patients should have failed at least two to three other migraine prophylactics unless contraindicated [43].

In general, OBTA is better tolerated than the oral migraine preventive drugs. A randomized double-blind trial comparing OBTA to topiramate in patients with chronic migraine in 2009 demonstrated the superior tolerability of OBTA. In the OBTA group, 7.7% discontinued because of adverse events, compared to 24.1% in the topiramate group [44]. In 2019, OBTA was compared with topiramate in a randomized parallel group open-label study. Only 1% of subjects in the OBTA group withdrew because of adverse events, compared to 42% in the topiramate group [45].

The main barriers to wider use of OBTA in chronic migraine are cost and the need for special training to administer it. Patient pain and discomfort during administration and the need to visit a health care professional every three months may be additional barriers, although the quarterly visits to the health professional without the need to take daily medication may enhance compliance with treatment.

Barriers to the use of OBTA, particularly cost, have important implications for patient care. Patients who depend on publicly funded drug plans, for example in the province of Ontario, Canada, face major restrictions in their access to OBTA that are not consistent with published evidence and good clinical care [46], and the same is no doubt true for many patients without drug insurance.

Although onabotulinumtoxinA has been used off-label in adolescents, a small RDBPC trial, which randomized 125 patients, was negative [47].

## 9. OnabotulinumtoxinA and the CGRP Antagonists

With the advent of monoclonal antibodies directed against CGRP or its receptor (CGRP-As), the landscape in chronic migraine prophylaxis has shifted. In many jurisdictions, the use of these newer therapies will also be limited by cost.

There have been no head-to-head trials between OBTA and the CGRP-As. The placebo controlled trials are not directly comparable, but in the pooled PREEMPT trial data, OBTA reduced headache hours per month by 39 h more than placebo at six months [21], compared, for example, to a reduction of 19 h per month more than placebo by erenumab at 3 months [48]. OBTA was also being compared to a much larger placebo response. In the PREEMPT trials, the reduction in headache hours per month from baseline by placebo was 80, versus 55 in the erenumab study. Galcanezumab reduced headache hours per month by 23 and 18 (depending on the dose) hours more than placebo during the first three months of therapy in a chronic migraine study [49]

Although follow up for several years of patients enrolled in an erenumab clinical trial has revealed no safety concerns [50], long-term safety remains an issue with the CGRP-As, as CRGP, which has many functions throughout the body, is blocked on a systemic level. In contrast, in the doses used in chronic migraine, OBTA has primarily local effects without concerns about systemic side effects.

On the other hand, the ease of administration of the CGRP-As with a single monthly subcutaneous injection or in some cases an injection every three months as compared to the complicated three-monthly injection paradigm for OBTA may be an advantage. This advantage can potentially make CGRP-A therapies more available to patients and more convenient for both the prescriber and the patient.

## 10. Other Headache Types

OBTA has been tried in many headache disorders in addition to chronic migraine, but a detailed discussion of these is beyond the scope of this review.

### 10.1. Trigeminal Autonomic Cephalalgias and Nummular Headache

A recent review concluded that the use of OBTA was reasonable for cluster headache, other trigeminal autonomic cephalalgias, and nummular headache when other therapies have failed, but this was based entirely on clinical experience, case series, and case reports [51].

### 10.2. Trigeminal Neuralgia

The use of BTA in trigeminal neuralgia is supported by four positive RDBPC trials, which included a total of 178 patients, although a recent meta-analysis suggested the quality of most of these trials was poor [52]. BTA is an option for patients if standard medications are not effective enough or not tolerated, or if surgery is not an option or unsuccessful.

### 10.3. Chronic Tension-Type Headache

A systematic review of OBTA in chronic tension-type headache found 11 RDBPC trials but concluded more research was needed. In contrast to positive results from non-randomized trials, the authors found “low evidence supporting any conclusions” in the RDBPC trials [53].

### 10.4. Persistent Headache Attributed to Whiplash

A systematic review in 2010 found three small randomized controlled trials (RCT) of BTA therapy in whiplash-associated disorder (WAD). Treatment usually involved injection of myofascial tender points. Two trials reported some positive results, but the reviewers concluded there was contradictory evidence regarding the effectiveness of BTA therapy during the chronic stage of WAD [54]. In 2011, a rigorous RDBPC trial done in patients with cervicogenic headache, almost half of whom had previous head or neck trauma, concluded that OBTA injected into the neck muscles was not beneficial [55].

In summary, the evidence for OBTA in persistent headache attributed to whiplash injury is minimal, but trials have involved small numbers of patients and injection protocols have varied from study to study.

### 10.5. Persistent Headache Attributed to Traumatic Injury to the Head

A review in 2016 reported no RCTs, but concluded that OBTA had shown promise in a few small studies [56]. More research is needed, but it would appear appropriate to use OBTA in patients with prior head trauma if their headache phenotype is that of chronic migraine.

### 10.6. Temporomandibular Disorder

Temporomandibular disorder (TMD) is a multifaceted disorder involving joints, muscles, and other tissues. OBTA is known to affect the intrafusal fibers of muscle spindles, and relaxation of these could reduce afferent input into the brainstem from spindles and reduce stretch reflexes and motor output [57]. In TMD, relevant actions of OBTA might involve effects on extrafusal motor nerve endings, nociceptive nerve endings, and muscle spindles.

A 2019 systematic review of BTA in the treatment of patients with TMD and/or bruxism included eleven controlled studies. It concluded that BTA seemed to lessen pain levels and could be considered once primary conservative treatment measures have proven ineffective [58].

A review from 2020 on the use of BTA in TMD included data from seven RCTs and 220 patients. The studies used heterogenous methodologies, and although most showed some benefit, others did not [59].

A 2017 review on BTA in sleep bruxism identified three RCTs and two uncontrolled trials. Small sample sizes, different injection techniques, and different outcome measures limited the conclusions, but there was some evidence for clinical benefit [60].

## 11. The Future

The development of more specific BTs that target the sensory neurons that mediate pain but do not affect motor neurons could be a major advance in chronic migraine therapy. This would likely allow use of an increased dosage for greater therapeutic benefit without unwanted local paralytic side effects.

The discovery or development of such a specific toxin may well be possible. All BTs consist of a 10 KDa heavy chain and a 5 KDa light chain linked by a disulfide bond. Over 40 different BT subtypes are known, and there are subtle differences in the effects they have on neuronal processes [26]. The heavy chain contains a receptor binding domain through, which BT binds to the cell surface. Small differences in the amino acid sequence of the heavy chain could lead to a toxin that would enter sensory neurons but not motor neurons.

Similarly, subtle differences in the light chain can affect which intracellular proteins the toxin will disrupt. The light chains of BT A, C, and E cleave SNAP-25, while the light chains of BT B, D, F, and G cleave VAMP on the vesicles. BT C also cleaves syntaxin on the plasma membrane. A natural version of BT may exist, or one could be produced artificially that would affect neurotransmitter release and/or receptor trafficking to the cell membrane in sensory neurons but not in motor neurons. Engineering of BTs has been made possible by recombinant technologies, and some molecules with analgesic properties that do not target the neuromuscular junction have already been produced [61].

Administration of the toxin through the skin rather than by injection could enhance its use in chronic migraine. Research has already been done in the use of BTA by transdermal delivery using molecular enhancers, liposomes, and iontophoresis for cosmetic indications and hyperhidrosis [4].

More evidence to support preliminary observations that OBTA is effective in high-frequency episodic migraine [23] and that early treatment of chronic migraine leads to better outcomes [41] might eventually result in wider use of OBTA.

Further research may show that patients with severe chronic migraine may benefit more from combination therapy with a CGRP-A and OBTA than from either treatment alone. This is theoretically plausible as OBTA is thought to block release of CGRP and other peptides from the terminals of C-fiber nociceptors, thereby preventing or reversing peripheral sensitization, reducing afferent activity in the C-fibers, and eventually reducing central sensitization. The reduction in CGRP release may also reduce afferent neural activity in the meningeal CGRP receptor-containing A-delta pain fibers.

A limiting factor for the efficacy of OBTA may be our inability to deliver the toxin to many of the C-fibers, as it may be that only those C-fibers with collateral axons to extracranial tissues become exposed to OBTA injected extracranially.

The CGRP-As which either block the CGRP receptors on the A-delta fibers or target CGRP itself so that it can no longer activate the receptors may act primarily by preventing activation of the A-delta fibers and thereby reducing afferent input into the TCC. As a systemic medication, the CGRP-As would have widespread access to the A-delta nerve terminals, but they likely do not reduce afferent activity in the C-fibers. Therefore, a combination of OBTA and CGRP-A might result in a greater reduction in afferent input into the TCC than would result from either agent given alone [33]. There is already some evidence that the addition of erenumab to patients on stable OBTA therapy does provide additional benefit [62], but more research needs to be done. Combination therapy would be expensive, and although chronic migraine can be both distressing and disabling, cost might become a major issue.

## 12. Conclusions

It has been over ten years since OBTA was approved for therapy of chronic migraine, and its off-label use goes back much further. Given its mechanism of action, it is unlikely to be displaced to a major extent by the new monoclonal antibodies directed against CGRP or its receptor. If new types of BTs are discovered or created with retained effects on pain nerve fibers but without effects on motor nerve fibers, the use of BTs for chronic migraine might well increase. Many patients with chronic migraine do not respond adequately to any one current therapy. All current treatment modalities and new ones are badly needed to reduce migraine-related disability.

## Abbreviations

Botulinum toxin (BT), botulinum toxin type A (BTA), onabotulinumtoxinA (OBTA), randomized double-blind placebo-controlled (RDBPC), CGRP antagonist (including ligand binding antibodies) (CGRP-A), randomized controlled trial (RCT).
